# Crescentic Glomerulonephritis and Membranous Nephropathy: A Rare Overlap

**DOI:** 10.1155/2022/8292458

**Published:** 2022-06-24

**Authors:** Mohamedanwar Ghandour, Heba Osman, Samer Alkassis, Alix Charles, Kristina Zalewski, Jarrett Weinberger, Yahya Malik-Osman, Zeenat Y. Bhat

**Affiliations:** ^1^Department of Internal Medicine WSU SOM, Division of Nephrology Wayne State University, Detroit, MI, USA; ^2^Department of Internal Medicine/Pediatrics, Wayne State University/Detroit Medical Center, Detroit, MI, USA; ^3^Department of Internal Medicine WSU SOM, Wayne State University, Detroit, MI, USA

## Abstract

**Background:**

Membranous nephropathy (MN) is a disease that affects the basement membrane of the glomeruli of the kidney resulting in proteinuria. The concurrent incidence of vasculitic glomerulonephritis and MN in the same patient is unusual. Herein, we report a case with this unusual combination.

**Case:**

Our patient is a 53-year-old Hispanic male with a medical history of tobacco use, type 2 diabetes mellitus, and hypertension who presented with hematuria and was found to have nephrotic range proteinuria and renal impairment. Blood workup revealed positive ANCA serology, which led to a renal biopsy that showed crescentic vasculitis in addition to membranous nephropathy. The patient was started on intermittent hemodialysis (HD) and treated initially with intravenous (IV) pulse steroids; subsequently, oral prednisolone and IV cyclophosphamide were initiated. The patient remained HD dependent at the time of discharge with the resolution of hematuria. A follow-up with an outpatient nephrology clinic was arranged.

**Conclusion:**

Membranous nephropathy complicated by crescentic glomerulonephritis has a more aggressive clinical course and decline in renal function compared to MN alone which can lead to initiating renal replacement therapy. However, immunosuppressive drugs can result in significant improvement of renal function if started early enough.

## 1. Introduction

Membranous nephropathy (MN) is a common etiology of nephrotic syndrome, especially in on-diabetic adults. Histologically, it is characterized by a light microscopy pattern of basement membrane thickening with deposition of immunoglobulins and complement proteins [1]. MN can be either idiopathic or associated with an underlying disease such as systemic lupus nephritis, other autoimmune disorders, malignancies, or drugs such as nonsteroidal anti-inflammatory drugs (NSAIDs) [2–4]. On the other hand, antineutrophil cytoplasmic autoantibody (ANCA)-associated vasculitis (AAV) is a group of chronic inflammatory diseases affecting small and medium-sized vessels. Literature reports rare cases of vasculitic or crescentic glomerulonephritis in membranous nephropathy, except in those cases associated with systemic lupus erythematosus [5, 6]. Herein, we report a rare case of crescentic glomerulonephritis with membranous nephropathy. To our knowledge, the immunopathogenesis of this uncommon overlap of membranous nephropathy and crescentic glomerulonephritis is limited.

### 1.1. Case Presentation

Our patient is a 53-year-old Hispanic male with a medical history of tobacco use, type 2 diabetes mellitus, and HTN who presented to the emergency department with hematuria and diarrhea for 4 days. He additionally complained of symptoms of chronic sinusitis and epistaxis that occurred all year round. His medications at home included tramadol, celecoxib, glipizide, metformin, lisinopril, and metoprolol. Physical examination was only remarkable for 1+ pitting edema in his lower extremities bilaterally and dental caries. There were no signs of photosensitivity, malar rash, oral ulcers, lymphadenopathy, pericarditis, or sinus involvement. On admission, the patient was found to have a creatinine of 8.04 mg/dL (reference range 0.70–1.3 mg/dL). His baseline creatinine was 0.9 mg/dL less than eight months ago. His urinalysis revealed gross and microscopic hematuria, with RBC >100/HPF, 3+ protein, and no casts. A CT abdomen/pelvis was performed on admission which was unremarkable for any signs of obstruction; however, it revealed fibrotic changes in the lower lung bases. The urine protein creatinine ratio (UPCR) was 19.2 g/mg and low serum albumin was 2.7 gm/dL (reference range 3.5–5.7 gm/dL). ANCA antibody was positive; however, all viral markers (hepatitis B, C, syphilis EIA, and HIV), complement C3 and C4 levels, and autoimmune antibody profiles (ANA, rheumatoid factor, anti-ds DNA antibody, and antiglomerular basement membrane antibody) were all negative or within normal limits. The patient had high titers of myeloperoxidase (MPO) antibody. All other infectious workups such as urine culture and blood cultures were negative. CT of the lungs revealed interval worsening of fibrotic changes with honeycombing predominantly in the lung bases, suggesting usual interstitial pneumonia. Pulmonology was consulted and reported this was consistent with ANCA-associated lung involvement. Renal ultrasound showed normal kidney size with no hydronephrosis.

**Figure 1:**
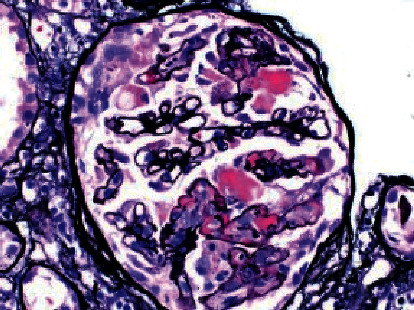
Light microscopy showing a glomerulus with focal segmental fibrinoid necrosis.

**Figure 2:**
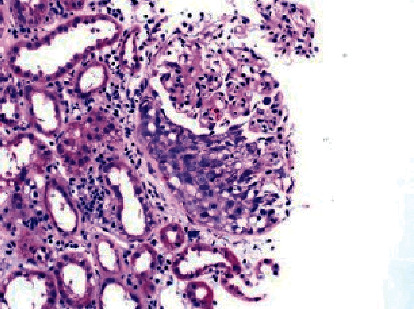
Light microscopy showing diffuse cellular crescent formation.

**Figure 3:**
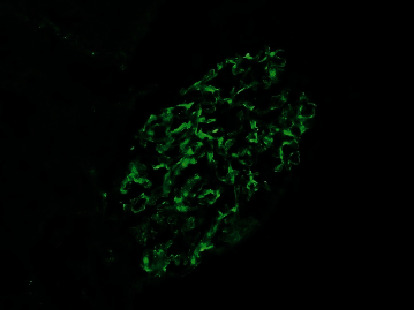
Immunofluorescence stained for IgA.

**Figure 4:**
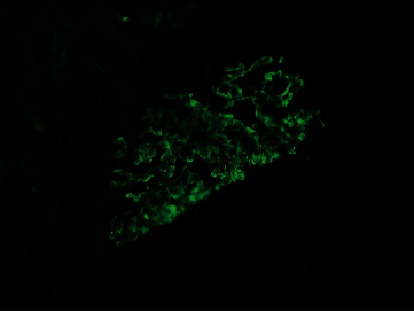
Immunofluorescence stained for IgG.

Renal biopsy was then performed which revealed membranous pattern glomerulonephritis with diffuse cellular crescents. There was moderate interstitial fibrosis, tubular atrophy with focal segmental fibrinoid necrosis (Figure 1). Light microscopy revealed 50% of sampled glomeruli had active cellular crescents (Figure 2). Immunohistochemical staining for phospholipase A2 receptor (PLA2R), thrombospondin type 1 domain-containing 7A (THSD7A), and neural epidermal growth factor-like 1 were negative. Immunofluorescence revealed granular capillary wall and mesangial staining for IgA (3+) and IgG (3+) (Figures 3 and 4). All the other stains were negative. Electron microscopy revealed extracapillary hypercellularity and segmental thickening in the glomerular capillary basement membranes with severe effacement of podocyte foot processes. There was no significant mesangial hypercellularity. Global subepithelial immune-type electron-dense deposits were identified, consistent with a membranous pattern with occasional intramembranous deposits.

Throughout his stay, the patient's creatinine continued to rise for which intermittent hemodialysis was initiated. The patient was also started on pulse dose steroids followed by maintenance steroids and intravenous cyclophosphamide. The patient reported a resolution of hematuria after the initiation of steroids; however, patient remained dialysis-dependent. The patient was discharged in a stable condition and scheduled for follow-up with the outpatient nephrology clinic.

## 2. Discussion

The primary antibodies identified in AAV are proteinase 3 (PR3) and myeloperoxidase (MPO)-ANCA antibodies. These autoantibodies are hallmarks of AAV, with PR3‐ANCA being the most common in GPA, while MPO‐ANCA is the most common in MPA. However, the association of vasculitic glomerulonephritis with membranous nephropathy is rare, estimated to be <5% of all membranous glomerulonephritis (MGN) cases, and is reported only in a handful of times in the literature [1]. Many concomitant MGN and AAV cases are also diagnosed together at presentation (Table 1). Our case had positive myeloperoxidase (MPO)-ANCA antibodies. The histological pattern of MGN is characterized by the formation of subepithelial immune complex deposits with changes to the glomerular basement membrane. Primary MGN occurs in approximately 75% of MGN cases and is caused by circulating anti-PLA2R antibodies against the podocyte antigens. In 25% of cases, secondary MGN occurs and is associated with hepatitis B, autoimmune diseases, thyroiditis, malignancies, and certain drug use such as nonsteroidal anti-inflammatory drugs (NSAIDs) [2].

Microscopic hematuria, proteinuria, and acute kidney failure are all common presentations of MGN associated with ANCA vasculitis [3, 4]. In a case series of 15 patients reported by Nikolopoulou et al., microscopic hematuria was detected in all of them except for one who was anuric. Moreover, proteinuria was also noticed with a median UPCR of 819.5 mg/mmol (reference range 88–5600) [3]. The reported case presented with UPCR was 19.2 g/day. Extrarenal symptoms are also common including fatigue, arthralgias, and malaise. A literature review done by Balafa et al., which included 38 patients with ANCA-positive MGN and 30 patients with ANCA-negative MGN, revealed that vasculitis symptoms predominated in the first group, but were absent in the second one [5].

The renal function at presentation along with the presence of antibodies may predict the clinical outcome. However, prognosis is variable with 40% of patients progressing to end-stage renal disease [3]. Patients with MGN and ANCA-associated NCGN are likely to have heavier proteinuria and a worse prognosis than patients with ANCA-associated NCGN alone [4]. In addition to higher levels of urinary protein excretion and decreased eGFR at the time of renal biopsy being risk factors for poor prognosis in MGN, crescent formation was reported to be another risk factor for worse renal outcome [6].

Prompt aggressive treatment can lead to stabilization of renal function and a significant decline in proteinuria. Treatment typically involves a combination of immunosuppressive agents such as steroids and cyclophosphamide and/or rituximab. Nasr et al.'s case series of 14 patients revealed that 5 out of 14 patients progressed to ESRD despite treatment; however, ANCA testing was repeated after treatment in 7 patients and revealed that 5 patients were seronegative [3]. In Nikolopolou et al.'s case series, 9 patients had stabilization or improvement of renal function, 6 had progressed to ESRD, and 4 died in the median 72-month follow-up period [4]. All the patients were treated with steroids, 10 with cyclophosphamide and 6 with rituximab [4]. There is currently not enough evidence to guide treatment when deciding between the addition of cyclophosphamide versus rituximab.

## 3. Conclusion

Our case of a 53-year-old man who was found to have both ANCA-associated GN and MGN with crescents on kidney biopsy represents a rare dual glomerulopathy. An overlap between MN and ANCA is rare and might require a histological diagnosis. Presentation with proteinuria, microscopic hematuria, and acute kidney injury should prompt treatment for rapidly progressive glomerulonephritis to prevent poor clinical outcomes and progression to end-stage renal disease.

## Figures and Tables

**Table 1:** Clinical details of patients with membranous nephropathy and concomitant vasculitic glomerulonephritis.

Title/authors	Patient (age/sex)	PLA2R	ANCA by IF	ANCA specificity by ELISA	Treatment	Prognosis
Membranous nephropathy with proteinase 3-ANCA-associated vasculitis successfully treated with rituximab; Shun Yoshida, Shunichiro Hanai, Daiki Nakagomi, Kei Kobayashi, Kazuya Takahashi, Fumihiko Furuya [7]	73/F			PR3	Oral prednisolone and IV rituximab	Renal function immediately improved, along with symptoms and urinalysis abnormalities
Association of vasculitis glomerulonephritis with membranous nephropathy: A report of 10 cases; Tse WY, Howie AJ, Adu D, Savage CO, Richards NT, Wheeler DC, Michael J [1]	10 patients: 9 males/1 female, 30–70 years					Renal function improved in 3 patients; 2 patients required RRT; 3 patients died: one of systemic vasculitis and 2 of sepsis
	30/M		C-ANCA		Oral prednisolone and cyclophosphamide	Stable renal function. Complication: squamous lung CA 10 years after presentation
	39/M		Negative		Oral prednisolone and cyclophosphamide	Recovery. Complication: steroid-induced DM. Duration of follow-up: 7 months
	41/M		Negative		Oral prednisolone and cyclophosphamide	Stable renal function, duration of follow-up: 3 years
	58/F		Negative		Oral prednisolone and cyclophosphamide	Dialysis, duration of follow-up: 7 years
	63/M		NA		Oral prednisolone and azathioprine for 3 years, followed by prednisolone and cyclophosphamide	Death, duration of follow-up: 6 years
	64/M		P-ANCA		Oral prednisolone and azathioprine	Dialysis, duration of follow-up: 2 years
	65/M		P-ANCA		Oral prednisolone and cyclophosphamide	Recovery, duration of follow-up: 5 years
	65/M		Negative		Oral prednisolone and cyclophosphamide	Death (after 4 months)
	68/M		Negative		Oral prednisolone and cyclophosphamide	Death (after 2 months)
	70/M		C-ANCA		Oral prednisolone, cyclophosphamide, and plasma exchange	Recovery (duration of follow-up:4 years)
Membranous glomerulonephritis with ANCA-associated necrotizing and crescentic glomerulonephritis; Samih H. Nasr, Samar M. Said, Anthony M. Valeri, Michael B. Stokes, Naveed N. Masani, Vivette D. D'Agati, and Glen S. Markowitz [4]	64/M		C-ANCA	NA	Prednisone and cyclophosphamide	Resolution of pulmonary lesions, normalization of Cr, diminution of proteinuria
	68/F		NA	MPO	Prednisone	Normalization of Cr
	47/F		P-ANCA	MPO	Methylprednisolone and then prednisone and cyclophosphamide	Diminution of proteinuria and disappearance of crescents (on repeat bx)
	67/M		P-ANCA	MPO	Prednisone and azathioprine	Dialysis
	69/M		P-ANCA	MPO	Prednisone and cyclophosphamide	Normalization of Cr
	68/F		N/A	MPO	Prednisone and cyclophosphamide	Partial recovery
Co-occurrence of PLA2R-positive membranous nephropathy without crescents, and PR3-positive eosinophilic granulomatosis with polyangiitis; Yuexin Zhu, Qing Chang, Xiangyan Cao, Song Zheng, Peiling Li, Junjun Luan, Hua Zhou [8]	??	PLA2R positive	ANCA positive	PR3	Prednisone and cyclophosphamide	Recovery
Anti-neutrophil cytoplasmic antibody-positive eosinophilic granulomatosis with polyangiitis: can it cause membranous nephropathy? S B Mahmood, H Ahmad, J Wu, D Haselby, M M LeClaire, R Nasr [9]	63/F		P-ANCA	MPO	Rituximab	Recovery
Primary membranous nephropathy presenting with crescentic glomerulonephritis 25 years after initial presentation: A case report; David Massicotte-Azarniouch, Sean Barbour, Paula Blanco, Edward G Clark [10]	63/M	PLA2R positive	Negative ANCA	Negative	Prednisone and cyclophosphamide and then azathioprine for maintenance	Dialysis and then partial recovery
Anti-neutrophil cytoplasmic antibody-associated glomerulonephritis with detection of myeloperoxidase and phospholipase A2 receptor in membranous nephropathy lesions: report of two patients with microscopic polyangiitis; Tominaga K, Uchida T, Imakiire T et al. [11]	52/M	PLA2R positive	Not reported	MPO	Not reported	Not reported
	63/F	PLA2R positive	Not reported	MPO	Not reported	Not reported
Crescentic glomerulonephritis and membranous nephropathy: A rare coexistence; Olga Balafa, Rigas Kalaitzidis, Georgios Liapis, Sofia Xiromeriti, Fotios Zarzoulas, Georgios Baltatzis and Moses Elisaf [5]	58/M		p-ANCA		Prednisolone, cyclophosphamide, and plasmapheresis	Recovery, duration of follow-up: 3 months
Clinical and immunologic characteristics of patients with ANCA-associated glomerulonephritis combined with membranous nephropathy a retrospective cohort study in a single Chinese center; Zou, Rong; Liu, Gang; Cui, Zhao; Chen, Min; Zhao, Ming-Hui [12]	27 patients with ANCA-GN and characteristics of MN on renal biopsy	17 M and 10 F, with an age of 52.4 ± 17.7 years	25 p-ANCA positive; 2 c-ANCA positive	25 MPO positive and 2 PR3 positive	Prednisone and cyclophosphamide	11 of 27 (40.7%) died; 13 of 27 (48.1%) progressed to ESRD
	17 M and 10 F, with an age of 52.4 ± 17.7 years					ANCA-GN patients with MN had significantly poorer renal outcome (*P* = 0.021) and patients' survivals (*P* = 0.036) compared with the patients without MN
						No significant difference in causes of death between ANCA-GN patients with and without MN.
						Infection is the first cause of death in ANCA-GN patients with and without MN
Membranous nephropathy with crescents: A series of 19 cases; Erika F. Rodriguez, Samih H. Nasr, Christopher P. Larsen, Sanjeev Sethi, Mary E. Fidler, Lynn D. Cornell [13]	19 patients with ANCA and crescentic MN	38% PLA2R positive	All negative	All negative		
	No patient had positive anti-dsDNA, hep B and C, or HIV					
	22/M		Negative	Negative	Prednisone and cyclophosphamide and then cyclosporine	Recovery, duration of follow-up: 138 months
	76/F		Negative	Negative	Prednisone and cyclophosphamide	Partial recovery, duration of follow-up: 26 months
	80/F		Negative	Negative	Mycophenolate mofetil and prednisone	Recovery, duration of follow-up:6 months
	69/F		Negative	Negative	Prednisone	ESRD, duration of follow-up: 1.5 months
	57/M		Negative	Negative	Prednisone and cyclophosphamide orally with remission; then azathioprine; then prednisone and cyclophosphamide	Duration of follow-up: 56 months
	41/M		Negative	Negative	Prednisone and cyclophosphamide	Worsening renal function, duration of follow-up:5 months
	20/F		Negative	Negative	None	ESRD, duration of follow-up: 35 months
	17/F		Negative	Negative	Enalapril	Recovery, duration of follow-up: 3 months
	50/M		Negative	Negative	Prednisone, cyclophosphamide, and mycophenolate	Partial recovery, duration of follow-up: 16 months
	5/F		Negative	Negative	Prednisone and mycophenolate (no response at 6 m); then, prednisone and cyclosporine; then, prednisone and tacrolimus	Recovery, duration of follow-up: 32 months
	86/M		Negative	Negative	Prednisone	Partial recovery, duration of follow-up: 11 months
	64/M		Negative	Negative	Prednisone and cyclosporine	Worsening renal function, duration of follow-up: 2 months
	72/F		Negative	Negative	Prednisone and cyclophosphamide	Partial recovery, duration of follow-up: 27 months
	62/M		Negative	Negative	Unknown	Partial recovery, duration of follow-up: 19 months
	64/M		Negative	Negative	Prednisone and cyclosporine (no response at 2 m); then 4 doses rituximab; then mycophenolate and prednisone (no response)	ESRD, duration of follow-up: 11 months
	72/M		Negative	Negative	Losartan	Partial recovery, duration of follow-up: 9 months
	58/M		Negative	Negative	Prednisone and cyclophosphamide orally for 2 m; then azathioprine for 1 y	Partial recovery, duration of follow-up: 11 months
	70/F		Negative	Negative	Unknown	Dialysis within 1 month
	56/M		Negative	Negative	Prednisone and cyclophosphamide	Partial recovery, duration of follow-up: 3 months
A case of membranous glomerulonephritis with superimposed anti-neutrophil cytoplasmic antibody-associated rapidly progressive crescentic glomerulonephritis; Yoo Hyung Kim, Hae Ri Kim, Young Rok Ham, Jae Woong Jeon, Sarah Chung, Dae Eun Choi, Kang Wook Lee and Ki Ryang Na [14]	65/M	Not reported	p-ANCA	MPO	Methylprednisolone and cyclophosphamide	ESRD
Membranous glomerulonephritis with superimposed ANCA-associated vasculitis: Another case report; Antonio Granata, Fulvio Floccari [15]	67/M	Not reported	p-ANCA	Not reported	Methylprednisolone and cyclophosphamide	Partial recovery
